# Global longitudinal strain as a predictor of short-term effect of oral antiviral regimens on myocardium in Egyptian patients with chronic viral hepatitis C

**DOI:** 10.1186/s43044-020-00129-2

**Published:** 2021-01-09

**Authors:** Hazem Mohammad-Ali Farrag, Mina Samir Monir, Wael Soliman Abdel-Dayem, Hisham Abdel-Haleem Ali, Alaa Mohammad Ibrahim

**Affiliations:** 1 Cardiovascular Medicine Department, Faculty of Medicine, Minia University, Minia, Egypt; 2 Cardiology Department, Minia University Hospital, Minia, Egypt; 3 Tropical Medicine Department, Faculty of Medicine, Minia University, Minia, Egypt; 4 Internal Medicine Department, Faculty of Medicine, Minia University, Minia, Egypt

## Abstract

**Background:**

Hepatitis C virus (HCV) infection has been noted with various cardiovascular (CV) diseases, and patients with detected HCV-RNA had higher CV mortality than uninfected individuals. The new direct-acting antiviral drugs (DAA) proved to be more effective with fewer side effects compared to interferon in eradicating HCV, but their effect on myocardium is still questionable. In order to get some answers for such question, two-dimensional speckle tracking echocardiography (2D-STE) was studied before and after treatment with different DAA regimens in HCV patients with either mildly impaired or normal basic left ventricular ejection fraction (LVEF).

**Results:**

Global longitudinal strain (GLS) significantly worsened after finishing antiviral treatment in patients with basic impairment of LVEF (*n* = 100) and those with normal basic LVEF (*n* = 20) [*p* = 0.006 and 0.039, respectively]; also, segmental strain showed significant worsening of many segments. Such worsening was significantly more in those with basic impairment of LVEF compared to those with normal basic LVEF (*p* = 0.036). No significant difference was observed in GLS and segmental strain when classifying and comparing patients according to gender, presence of diabetes mellitus, hypertension, ischemic heart disease and established cardiac medications, or according to DAA regimen received.

**Conclusion:**

DAA may have a cardiotoxic effect that could be early detected by 2D-STE, which was more significant in patients with pre-treatment impairment of LVEF.

## Background

Hepatitis C virus (HCV) infection is a serious health problem in many developing African and Asian countries, with the highest prevalence in Egypt, mostly due to the previous mass parenteral anti-schistosomal treatments [1]. HCV was accused to be involved in many different forms of heart disease. And as the myocardium could be targeted by many types of viral infections, HCV infection has been noted in patients with myocarditis, cardiomyopathies, and in those with left ventricular (LV) diastolic dysfunction [2]. Several studies had shown that patients with detectable HCV-ribonucleic acid (RNA) had higher cardiovascular mortality than uninfected individuals, and those with undetectable HCV-RNA or sustained viral eradication may have a lower risk for CV disease [3, 4]. Recently, the use of new DAA regimens was proven to be more effective in the eradication of HCV than interferon with relatively fewer side effects, but their effect on the heart still questionable [5].

Two-dimensional (2D) speckle tracking echocardiography (STE) is a promising modern cardiac imaging modality that provides an important estimation of both systolic and diastolic function, ischemic myocardial affection, and many other pathophysiological processes, through calculating myocardial velocities and deformation parameters such as strain and strain rate [6].

In the current study, we evaluated the effect of different DAA regimens on myocardial function in chronic viral hepatitis C patients with either mildly impaired or normal pretreatment left ventricular ejection fraction (LVEF) using 2D-STE.

## Methods

This is an observational prospective comparative study that recruited 153 patients with chronic viral hepatitis C, who were candidate for receiving one of the commonly used DAA regimens in Egypt [sofosbuvir (SOF) and daclatasvir (DCV) **±** Ribavirin (RBV)] after receiving their medical records and treatment strategies from the hepatology outpatient clinic.

### Inclusion criteria

Patients with mildly impaired LV systolic function (LVEF = 40–50%, regardless the cause of impairment) and patients with normal LV systolic function (LVEF ≥ 55%) were included.

### Exclusion criteria

Patient with borderline LVEF (50-54%) or those with LVEF<40%, patients who were discovered to have any contraindication for DAA or discontinued their treatment course, and patients with atrial fibrillation (AF) -as difficult performance of STE due to beat-to-beat variability- were excluded.

The study protocol was approved by the institutional ethical committee and agreed with the “world medical association declaration of Helsinki”. After obtaining an assigned consent from every participating patient, they were asked for history of cardiac and hepatic diseases, cardiac medication, other co-morbidities like diabetes mellitus (DM), arterial hypertension, and/or ischemic heart diseases (IHD). They were also asked to report any emerging cardiac symptoms during treatment course. Patients were subjected to full cardiac examination and 12-lead surface electrocardiogram (ECG) before and during treatment course for detecting any emerging cardiac signs or EGC changes that might interfere with treatment continuation or 2D-STE evaluation like AF, bradycardias, prolonged QT interval, and/or ST-T wave changes. Transthoracic echocardiography was performed before and after finishing treatment regimen (3 months later) by the same operator, using SIEMENS ACUSON SC 2000 ultrasound machine with its dedicated (4 V1) probe (*2013, Siemens Medical Solutions USA, Inc*.). All measurements were performed according to the recommendations of the American Society of Echocardiography/European Association of Cardiovascular Imaging (ASE/EACVI) guidelines [7]. LVEF was estimated by Teichholz formula [8], and resting wall motion abnormalities were analyzed.

Two-dimensional STE was performed from the apical views (apical four-chamber, two-chamber, and long-axis). The standard grayscale images were obtained at a frame rate of 50–90 frames/second during three consecutive cardiac cycles, and software package (*velocity vector imaging VB10D, Siemens*) was used for offline analysis by two independent examiners. The 2D strain was measured by tracing the endocardial contour at end-diastolic frame in each view, then the software allowed to track the contour on subsequent frames automatically, and manual correction of the contour was also done to ensure optimal tracking. Longitudinal strain was analyzed for sixteen LV segments [namely: basal (anterior, anteroseptal, anterolateral, inferior, inferoseptal, and inferolateral), mid (anterior, anteroseptal, anterolateral, inferior, inferoseptal, and inferolateral), apical (anterior, inferior, septal, and lateral) segments], and GLS values was measured as the average of segmental values [9].

*Statistical analysis* was performed using IBM statistical package for the social sciences (SPSS) software—version 20 for Windows (*SPSS Inc., Chicago, Illinois, USA*). Data were expressed as mean ± standard deviation (SD) for quantitative parametric measures or median in quantitative non-parametric measures, in addition to both number and percentage for categorized data. Student’s *t* test was used for comparison between independent groups for parametric data, and Mann-Whitney test for non-parametric quantitative. The paired *t* test was used to compare paired parametric data. Chi-square (*χ*^*2*^) *test* or Fisher’s exact test (if more than 20% of expected counts less than 5) were used to compare qualitative data. Statistical significance was defined as a probability level of *p* < 0.05.

## Results

From the 153 recruited patients, 120 patients were included (33 patients were excluded: 4 patients had AF and 1 patient developed a new AF during the treatment course, 10 patients had pretreatment LVEF < 40%, 7 patients stopped treatment due to noncardiac causes, and 11 patients were missed during the study course). The mean age of the study population was 57.8 ± 6.4 years, 30.8% were females, 43.3% were diabetics, 30.8% were hypertensives, 28.3% had documented IHD [history of previous hospitalization with acute coronary syndrome, previous positive non-invasive testing for coronary artery disease (CAD), definite regional wall motion abnormalities which were detected at basic resting echocardiography, atherosclerotic CAD by previous coronary angiography, and/or history of previous coronary revascularization], and 29.2% were pre-medicated with chronic cardiac treatment [renin-angiotensin-aldosterone-system blockers, beta blockers, antiplatelets, and/or statins]. About 73% of the studied population were scheduled to receive dual DAA (SOF and DCV), while about 27% were scheduled to receive triple DAA (SOF, DCV, and RBV).

The study cohort was divided into group I: those with basic LVEF = 40–50% (*n* = 100) and group II: those with basic LVEF ≥ 55% (*n* = 20). No significant difference was observed between both groups regarding age, gender, presence of DM, hypertension, and/or documented IHD, pre-medication with chronic cardiac treatments, and the DAA regimen used (Table 1).

**Table 1:** Demographic and clinical data, received DAA regimen at study groups

	Group I (***n*** = 100)	Group II (***n*** = 20)	***p*** value
**Age in years**			
Mean ± SD (range)	58.6 ± 6.5 (45–77)	56.2 ± 6.7 (45–66)	0.139
**Gender**, no (%)			
**Male**	28 (28%)	9 (45%)	
**Female**	72 (72%)	11 (55%)	0.133
**Diabetics**, no (%)	47 (47%)	5 (25%)	0.07
**Hypertensives**, no (%)	32(32%)	5 (25%)	0.536
**Documented IHD**, no (%)	30 (30%)	4 (20%)	0.265
**Cardiac premedication**, no (%)	31 (31%)	4 (20%)	0.241
**Pretreatment LVEF**%, no (%)	46.9 ± 2.5 (40-50)	65.05 ± 4.4 (58-73)	**< 0.001**
**DAA regimen**, no (%)			
**Dual**	73(73%)	15(75%)	
**Triple**	27(27%)	5(25%)	0.854

*DAA* direct-acting antiviral drugs, *IHD* ischemic heart disease, *LVEF%* left ventricular ejection fraction percent

There was no significant alteration in patients’ symptoms, signs, and ECG with treatment. Besides, there was no significant alteration in other echocardiographic parameters regarding chambers volumes, dimensions, and ventricular diastolic functions.

**Fig. 1:**
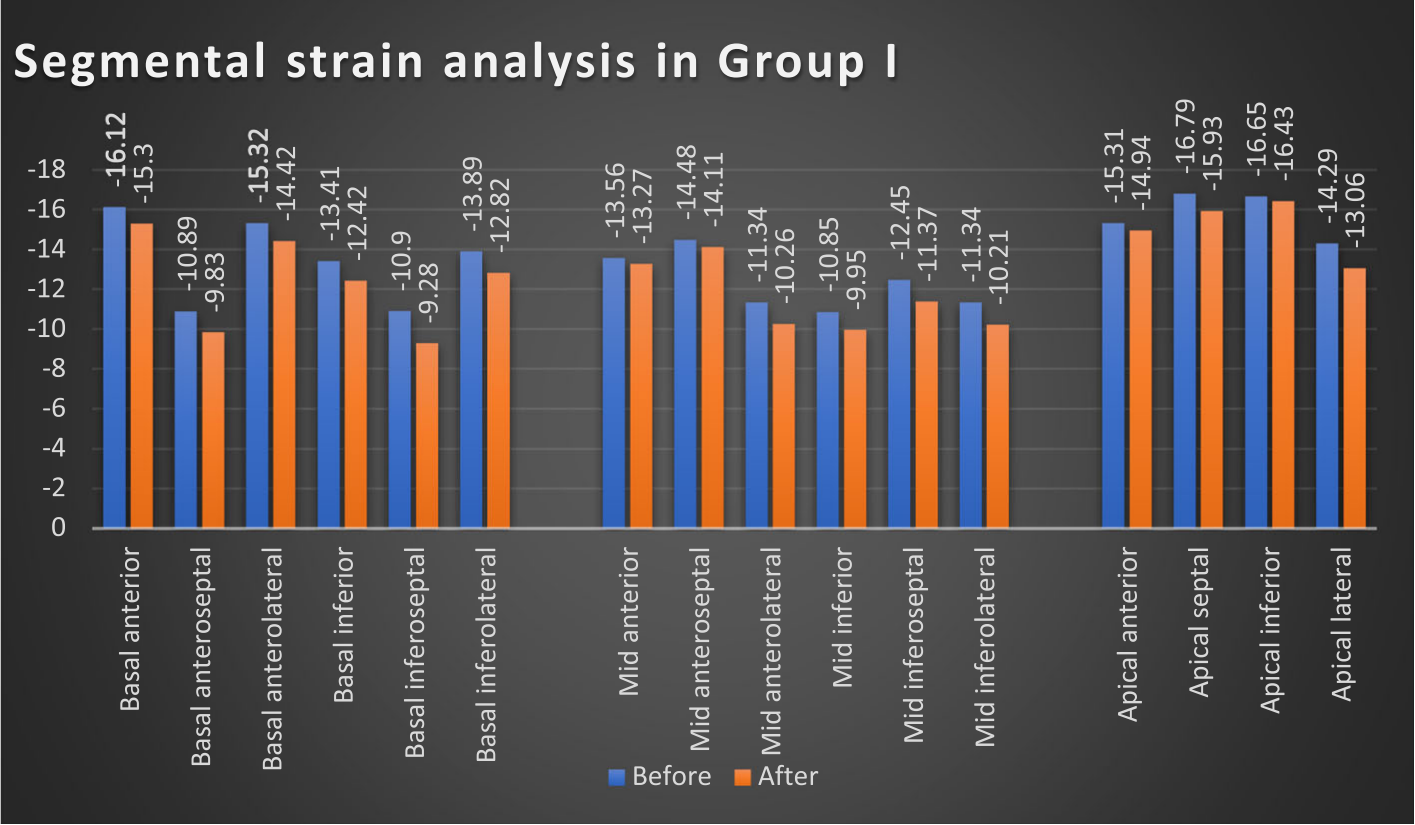
Segmental strain analysis before and after receiving antiviral treatment in group I

**Fig. 2:**
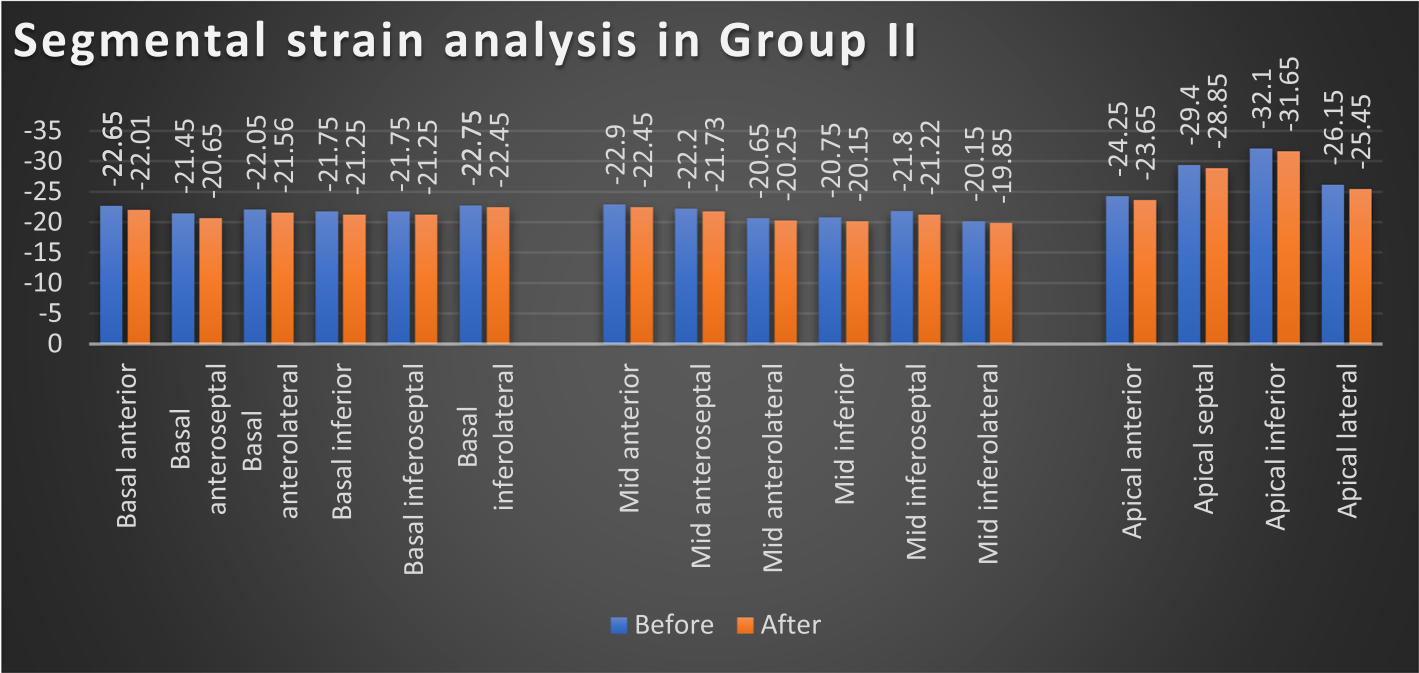
Segmental strain analysis before and after receiving antiviral treatment in group II

**Fig. 3:**
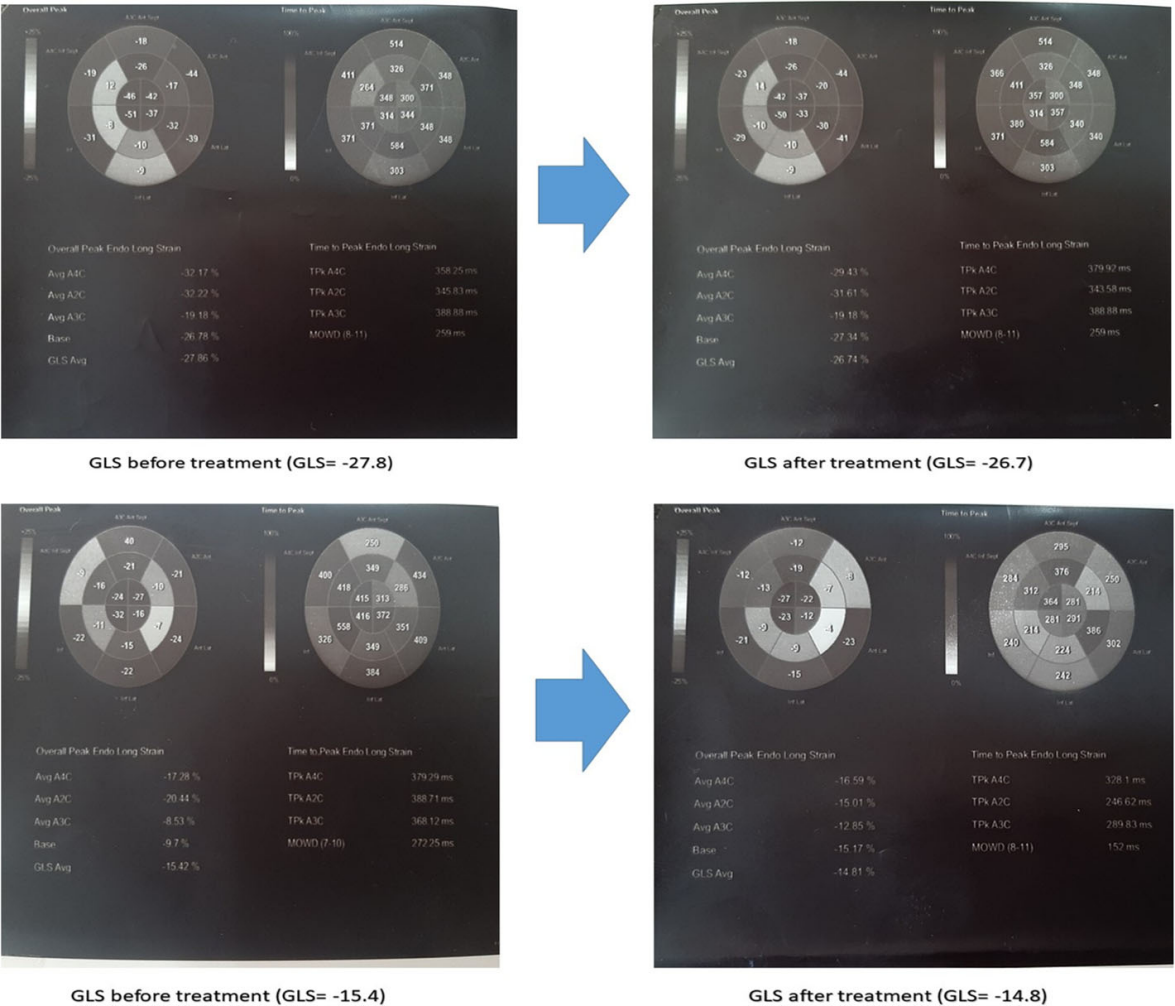
Global longitudinal strain (GLS) and segmental strain analysis before and after receiving antiviral treatment in real studied cases. Upper panel images show change in changes in GLS and segmental strain analysis at case no. 15 of group I, GLS = − 27.8 before receiving antiviral treatment (left image), which worsened to − 26.7 by receiving treatment (right image). Lower panel images show change in changes in GLS and segmental strain analysis at case no. 3 of group II, GLS = − 15.4 before receiving antiviral treatment (left image), which worsened to − 14.8 by receiving treatment (right image)

There was a statistically significant difference regarding 2D-STE results in group I patients before and after receiving antiviral regimen, with significant worsening of GLS values *(p = 0.003)* and segmental strain [except for apical anterior and inferior segments, mid anterior, and anteroseptal segments] after receiving DAA regimens. Meanwhile, such difference was not observed regarding LVEF in the same group (*p = 0.06*) (Table 2, Figs. 1 and 3). Similar pattern was obtained in group II, with significant worsening of GLS values (*p = 0.039*) and segmental strain [except for basal inferolateral segment, mid inferolateral, and anterolateral segments], while no significant difference was observed regarding LVEF in the same group (*p = 0.089*) (Table 2, Figs. 2 and 3).

**Table 2:** The GLS, segmental strain, and estimated LVEF% in group I and group II before and after receiving antiviral treatment at each group

	Group I (***n*** = 100)			Group II (***n*** = 20)		
	Before	After	***p*** value	Before	After	***p*** value
**LVEF**%	47.69 ± 2.54	47.01 ± 2.53	0.06	63.7 ± 3.96	63.29 ± 3.91	0.089
**GLS**	− 13.86 ± 0.83	− 12.83 ± 0.87	**0.003**	− 25.09 ± 2.04	− 24.41 ± 2.05	**0.039**
**Basal anterior**	− 16.12 ± 5.8	− 15.3 ± 5.81	**0.006**	− 22.65 ± 3.88	− 22.01 ± 4.48	**0.023**
**Basal anteroseptal**	− 10.89 ± 3.89	− 9.83 ± 2.92	**0.002**	− 21.45 ± 4.26	− 20.65 ± 3.99	**0.019**
**Basal anterolateral**	− 15.32 ± 6.41	− 14.42 ± 5.1	**0.006**	− 22.05 ± 4.5	− 21.56 ± 4	**0.033**
**Basal inferior**	− 13.41 ± 7.26	− 12.42 ± 5.45	**0.002**	− 21.75 ± 5.19	− 21.25 ± 5.13	**0.042**
**Basal inferoseptal**	− 10.9 ± 5.44	− 9.28 ± 3.38	**0.002**	− 21.75 ± 9.32	− 21.25 ± 7.61	**0.043**
**Basal inferolateral**	− 13.89 ± 5.22	− 12.82 ± 6.25	**0.001**	− 22.75 ± 5.72	− 22.45 ± 7.35	0.066
**Mid anterior**	− 13.56 ± 5.77	− 13.27 ± 6	0.085	− 22.9 ± 5.28	− 22.45 ± 4.95	**0.03**
**Mid anteroseptal**	− 14.48 ± 6.43	− 14.11 ± 5.77	0.075	− 22.2 ± 4.29	− 21.73 ± 5.06	**0.03**
**Mid anterolateral**	− 11.34 ± 3.32	− 10.26 ± 3.3	**0.001**	− 20.65 ± 3.25	− 20.25 ± 4.44	0.059
**Mid inferior**	− 10.85 ± 5.85	− 9.95 ± 3.97	**0.006**	− 20.75 ± 4.28	− 20.15 ± 5.42	**0.022**
**Mid inferoseptal**	− 12.45 ± 4.14	− 11.37 ± 4.59	**0.001**	− 21.8 ± 4.82	− 21.22 ± 4.12	**0.021**
**Mid inferolateral**	− 11.34 ± 5.17	− 10.21 ± 4.27	**0.004**	− 20.15 ± 4.42	− 19.85 ± 4.49	0.062
**Apical anterior**	− 15.31 ± 7.19	− 14.94 ± 9.42	0.080	− 24.25 ± 4.83	− 23.65 ± 6.21	**0.033**
**Apical septal**	− 16.79 ± 5.19	− 15.93 ± 9.39	**0.011**	− 29.4 ± 6.17	− 28.85 ± 6.41	**0.045**
**Apical inferior**	− 16.65 ± 6.78	− 16.43 ± 8.14	0.06	− 32.1 ± 7.98	− 31.65 ± 7.07	**0.016**
**Apical lateral**	− 14.29 ± 5.74	− 13.06 ± 6.8	**0.007**	− 26.15 ± 6.12	− 25.45 ± 5.98	**0.027**

*LVEF%* left ventricular ejection fraction percent, *GLS* global longitudinal strain

When comparing the absolute changes that happened regarding 2D-STE results by receiving DAA regimens between both groups, the degree of worsening in GLS was found to be more significant in group I compared to group II (*p = 0.036*), and similar pattern was observed in segmental strain of all analyzed segments, while change in LVEF did not follow the same pattern (*p = 0.184*) (Table 3).

**Table 3:** Absolute changes in GLS, segmental strain and estimated LVEF% after receiving antiviral treatment between studied groups

	Group I (***n*** = 100)	Group II (***n*** = 20)	***p*** value
**LVEF%**	0.68 ± 0.98	0.53 ± 0.4	0.184
**GLS**	1.13 ± 0.21	0.47 ± 0.24	**0.036**
**Basal anterior**	1.12 ± 8.06	0.65 ± 3	**0.031**
**Basal anteroseptal**	1.06 ± 3.32	0.58 ± 2.35	**0.014**
**Basal anterolateral**	1.3 ± 6.04	0.75 ± 3.65	**0.045**
**Basal inferior**	1.39 ± 7.34	0.6 ± 4.05	**0.039**
**Basal inferoseptal**	1.22 ± 4.98	0.59 ± 5.77	**0.028**
**Basal inferolateral**	1.07 ± 5.81	0.38 ± 4.01	**0.016**
**Mid anterior**	1.29 ± 7.41	0.55 ± 3.12	**0.022**
**Mid anteroseptal**	1.17 ± 9.84	0.4 ± 6	**0.035**
**Mid anterolateral**	1.08 ± 3.86	0.48 ± 4.85	**0.034**
**Mid inferior**	1.15 ± 6.17	0.6 ± 3.78	**0.042**
**Mid inferoseptal**	1.18 ± 5.42	0.53 ± 4.11	**0.024**
**Mid inferolateral**	1.03 ± 6.14	0.4 ± 4.63	**0.025**
**Apical anterior**	1.07 ± 12.83	0.45 ± 4.77	**0.037**
**Apical septal**	1.06 ± 7.18	0.51 ± 4.25	**0.046**
**Apical inferior**	1.12 ± 8.69	0.45 ± 2.87	**0.036**
**Apical lateral**	1.14 ± 8.83	0.35 ± 4.39	**0.025**

*LVEF%* left ventricular ejection fraction percent, *GLS* global longitudinal strain

No significant difference was observed regarding the absolute changes that happened regarding GLS by receiving DAA regimens, when classifying and comparing the studied population according to the presence or absence of DM (*p = 0.108*), hypertension (*p = 0.957*), IHD (*p = 0.943*), or cardiac pre-medication (*p* = *0.337*). Also, the absolute changes that occurred in segmental strain of the 16-segments examined and LVEF did not show any significant differences.

Moreover, when comparing the studied population according to the type DAA regimen received (dual or triple), no significant difference was observed in absolute changes that happened with treatment regarding segmental strain of all segments examined, GLS (*p = 0.257*) and LVEF (*p* = *0.176*) (Table 4). Furthermore, no significant correlation was observed between age and GLS in the studied population (*r = 0.058, p = 0.532*).

**Table 4:** Absolute changes in GLS, segmental strain and estimated LVEF% after receiving antiviral treatment in dual and triple DAA regimen populations

	Dual DAA (***n*** = 88)	Triple DAA (***n*** = 32)	***p*** value
**EF**	0.71 ± 1.04	0.69 ± 0.87	0.176
**GLS**	1.07 ± 0.32	1.02 ± 0.15	0.257
**Basal anterior**	1.12 ± 8.06	1.02 ± 8.06	0.952
**Basal anteroseptal**	1.06 ± 3.32	1.26 ± 3.32	0.881
**Basal anterolateral**	1.3 ± 6.04	1.28 ± 6.04	0.466
**Basal inferior**	1.39 ± 7.34	1.29 ± 7.34	0.681
**Basal inferoseptal**	1.22 ± 4.98	1.25 ± 4.98	0.245
**Basal inferolateral**	1.07 ± 5.81	1.17 ± 5.81	0.727
**Mid anterior**	1.29 ± 7.41	1.23 ± 7.41	0.483
**Mid anteroseptal**	1.17 ± 9.84	1.12 ± 9.84	0.858
**Mid anterolateral**	1.08 ± 3.86	1.18 ± 3.86	0.230
**Mid inferior**	1.15 ± 6.17	1.12 ± 6.17	0.733
**Mid inferoseptal**	1.18 ± 5.42	1.08 ± 5.42	0.996
**Mid inferolateral**	1.03 ± 6.14	1.01 ± 6.14	0.365
**Apical anterior**	1.17 ± 12.83	1.07 ± 12.83	0.365
**Apical septal**	0.71 ± 1.04	0.69 ± 0.87	0.176
**Apical inferior**	1.07 ± 0.32	1.02 ± 0.15	0.257
**Apical lateral**	1.12 ± 8.06	1.02 ± 8.06	0.952

*LVEF%* left ventricular ejection fraction percent, *GLS* global longitudinal strain, *DAA* direct-acting antiviral drugs

## Discussion

HCV infection is a persistent hepatic viral infection that constitutes a considerable health dilemma. It is estimated that more than 170 million individuals worldwide are infected, and 25% of them are suffering from its complications in form of liver cirrhosis, hepatocellular carcinoma, and/or hepatic cell failure [10]. In Egypt, HCV is of significant concern, whereas about 12% of the population is infected [11]. Treatment for HCV infection was basically evolved from interferon-based therapy to oral DAA agents, yet some safety concerns have arisen, and its possible cardiac toxicity is still under investigation [10].

The 2D-STE has recently emerged as a technique for evaluating LV systolic and diastolic functions. It measures the displacement of speckles on the 2D images, representing the myocardial deformation rather than volumetric change as seen by traditional LVEF estimation methods [12]. The GLS obtained by 2D-STE has previously been demonstrated to be of prognostic value in patients having a wide array of cardiac diseases [13]. Several studies supported the STE prognostic value in patients with heart failure (HF), as GLS added an incremental value to LVEF in prediction of adverse outcomes, providing an interesting tool for improving risk stratification in chronic systolic HF. Besides, it was also considered as an independent predictor of all-cause mortality in such patients [14–16].

So, out of this importance of GLS in prediction and prognosis, the current study was performed using GLS in an attempt to predict the possible effect of DAA regimens on the myocardium. The study included 120 HCV patients, who were scheduled for receiving oral DAA regimens. The study cohort was divided into two groups according to their pre-treatment LVEF: group I (*n* = 100) included patients with mildly impaired LV systolic function regardless of the cause [LVEF = 40–50%), group II (*n* = 20) included patients with normal systolic function [LVEF ≥ 55%]. The 2D-STE was obtained for all studied patients before starting antiviral treatment and after finishing the 3-month treatment course.

No significant alteration in patients’ symptoms, signs, ECG, and other echocardiographic parameters was observed with treatment, which was in agreement with findings that obtained by Biomy et al. who studied 170 HCV patients received different of antiviral regimens and were followed-up for 1 year. It showed that neither major cardiac symptoms (dyspnea, orthopnea, and chest pain) nor ECG changes were developed during the study [17].

The important finding of our study was that the LV function—assessed by 2D-STE, through estimating the GLS—got worse in all the studied population after finishing the DAA regimens, whatever the basic LVEF was normal or mildly impaired (that did not show any significant change with treatment). Such findings agreed with those of Mazzitelli et al. who studied 71 HCV patients with the mean pre-treatment LVEF = 56.7%, and the mean GLS = − 20.9. Patients received SOF-based regimens and were followed-up for 6 months. The study showed that GLS was significantly worsened in the overall population who received SOF-based regimens, while LVEF did not significantly change. Moreover, GLS continued to worsen after stopping DAA, possibly indicating their prolonged cardiotoxic effect [18].

The possible cardiac toxicity of DAA drugs are supported by the data from Ahmed et al., which showed that treatment with BMS-986094 [a nucleotide analog of HCV nonstructural 5B-polymerase-inhibitor in interferon-free combinations with DCV and RBV] was stopped after a young male patient experienced rapidly progressive HF and passed away, and 41.2% of patients had some evidence of cardiac dysfunction, with a modest elevation of plasma levels of brain natriuretic peptide (BNP)—the sensitive marker of myocardial stress—in the majority of cases. The pathological myocardial analysis of that index case showed profound myocyte injury with very limited areas of necrosis and no appreciable infiltration of polymorph nuclear cells [19]. This could explain the significantly elevated creatinine phosphokinase-mb subtype (CKmb), which was reported by El-Adawy et al. who studied patients receiving (SOF and DCV) or (SOF, DCV, and RBV) regimens [20].

Although the actual potential mechanisms of certain DAAs (as BMS-986094 and IDX-14184) related cardiotoxicity remains unclear, series of non-clinical in vitro and in vivo studies showed that it may be related to some potential changes in cardiac energy utilization rather than being a direct mitochondrial toxicant [21]. Further investigations for the potential mechanisms of cardiac dysfunction and its relevance to other DAA drugs are still needed.

To the best of our knowledge, this was the first study to investigate the effect of different DAA regimens in patients with impaired LV systolic function, and significantly more worsening in the GLS and the segmental strain was observed in such patients compared to those with normal basic LVEF. This could be explained by the fact that patients with impaired LV systolic function had important modification in their contractile proteins, as proteolysis of the myofilaments (myocytolysis) and reduction in the volume of myofibrils per cell, also the alteration in the expression and/or activity of myofilament regulatory proteins, that is proposed to decrease the contractile function in HF [22]. And based on the previously mentioned microscopic analysis of the myocardium in index case which was reported by Ahmed et al., the majority of the myocardium of both ventricles showed diffuse thinning and elongation of cardiac myocytes associated with fine interstitial fibrosis and marked interstitial edema, which is consistent with toxic cardiomyopathy [19]. This may explain why patients with impaired LVEF were more liable to significant myocardial injury by DAA than those with normal LVEF, which was early detected by 2D-STE.

Another important finding from the current study was that such effect of DAA regarding GLS, segmental strain and LVEF were not associated to a specific antiviral regimen, and no significant difference was observed when comparing the study population according to the type of received DAA regimens [i.e., dual (SOV + DCV) or triple (SOV + DCV + RBV)]. This finding was in agreement with the recent results by Ibrahim et al., who studied 2D-echo and STE in 100 HCV patients before and after finishing DAA regimens, and classified them into two groups according to the presence of liver cirrhosis and the type of DAA regimen received [50 non-cirrhotic patients who received dual therapy (SOF and DCV), and 50 cirrhotic patients who received triple therapy (SOF, DCV, and RBV)]. They found no significant difference regarding LV volumes, LVEF, and LV strain between both groups by receiving both regimens [23].

The effect of different DAA regimens was also investigated by El-Adawy et al. using cardiac magnetic resonance (CMR), who studied 390 patients with chronic HCV infection and received different DAA regimens. The study cohort was divided into four groups according to the treatment regimen [Group A was treated with ledipasvir (LED) and SOF, group B was treated with simeprevir (SIM) and SOF, group C was treated with SOF and DCV, and group D was treated with SOF, DCV, and RBV]. CMR was done before, during and after 6 months of treatment. They reported that some patients who received regimens including SIM or DCV had expressed edema in CMR and different degrees of cardiomyopathy, with elevation of cardiac enzymes (CKmb), denoting their possible cytotoxic effect. Interestingly, those changes were fully reversible at the 6-month follow-up without leaving permanent cardiac damage [20].

Furthermore, no significant difference was observed in the current study regarding the absolute changes that happened in GLS, segmental strain, and LVEF by receiving DAA regimens when classifying and comparing the studied population according to the presence or absence of DM, hypertension, IHD, or cardiac pre-medication.

These findings regarding DM were concordant to those of Mazzitelli et al. who showed no significant difference regarding GLS and LVEF between diabetic and non-diabetic patients who received DAA therapy [18]. Interestingly, an improvement in glycemic control during treatment with DAA therapy was reported by Dawood et al., who studied 460 patients with chronic HCV genotype-4-infection and type II DM, and showed that about 77% of patients with sustained virologic response after treatment with DAA achieved improvement in glycemic control by 3 months of treatment, and even about 26% of them needed to decrease their hypoglycemic agents’ dose [24]. Also, in a recent study by Mada et al., that studied 118 diabetic patients with HCV who received DAA regimens, a significant and meaningful reduction in hemoglobin A1C levels was observed if sustained virologic response had achieved [25]. Such results may give a possible explanation to the neutralizing effect of DM on myocardium with DAA therapy.

Our results regarding hypertension were also concordant to those of Mazzitelli et al., who showed no significant difference in GLS and LVEF regarding the presence of hypertension in their studied population [18]. This could be explained by the modest effect of DAA therapy on blood pressure (BP) as shown by Biomy et al., who reported that none of the studied 170-HCV patients on DAA drugs had developed abnormally elevated BP during and 6 months after finishing treatment, with a slight or insignificant change in mean BP at the study visits [17].

In a recent study by Zuo et al. [who studied 84 patients with dilated LV with impaired LVEF (< 45%) and were classified according to their coronary angiography into 41 ischemic cardiomyopathy (ICM) and 43 non-ischemic cardiomyopathy (NICM) patients], no significant difference was present in GLS between those with ICM and NICM, although the lower LVEF and relative apical sparing with basal worsening of LV longitudinal strain observed in NICM patients. They owed such strain features to diffuse myocardial lesions throughout the entire LV in NICM, while ICM had homogeneously reduced longitudinal strains but greater reduction of radial strain along the obstructed coronary arteries territories [26].

## Conclusion

Oral DAAs may have a cardiotoxic effect that could be detected by 2D-STE rather than traditional LVEF estimation. This effect was more obvious in those with basic impairment in LV systolic function, irrespective of age, gender, presence of DM, hypertension, IHD or cardiac pre-medication, and regardless of the type of DAA regimen used.

### Limitations

Lack of a longer-term follow-up of the LV function after finishing the DAA course, as the main target of the study was to assess the short-term effect of the drugs. No cardiac biomarkers were investigated, as our study focused mainly on assessing a possible affection of myocardial function by the commonly used DAA regimens. A larger scale of patients on different DAA regimens may be furtherly evaluated for confirming such possible cardiotoxic effect of those drugs.

## Data Availability

The datasets generated and/or analyzed during the current study are not publicly available as included in ongoing researches, but they are available from corresponding author on reasonable request.

## Abbreviations

2D: Two-dimensional
2D-STE: Two-dimensional speckle tracking echocardiography
AF: Atrial fibrillation
ASE: American Society of Echocardiography
BNP: Brain natriuretic peptide
BP: Blood pressure
CAD: Coronary artery disease
CKmb: Creatinine phosphokinase-mb subtype
CMR: Cardiac magnetic resonance
CV: Cardiovascular
DAA: Direct-acting antiviral drugs
DCV: Daclatasvir
DM: Diabetes mellitus
EACVI: European Association of Cardiovascular Imaging
ECG: Electrocardiogram
GLS: Global longitudinal strain
HCV: Hepatitis C virus
ICM: Ischemic cardiomyopathy
IHD: Ischemic heart disease
LED: Ledipasvir
LV: Left ventricle
LVEF: Left ventricle ejection fraction
NICM: Non-ischemic cardiomyopathy
RBV: Ribavirin
RNA: Ribonucleic acid
SIM: Simeprevir
SOF: Sofosbuvir
STE: Speckle tracking echocardiography

## References

[1] Matsumori A, Shimada T, Chapman N (2006). Myocarditis and heart failure associated with hepatitis C virus infection. J Card Fail.

[2] Danesh J, Collins R, Peto R (1997). Chronic infections and coronary heart disease. Lancet.

[3] Lee MH, Yang HI, Lu SN (2012). Chronic hepatitis C virus i;fection increases mortality from hepatic and extrahepatic diseases: a community-based long-term prospective study. J Infect Dis.

[4] Hsu C-S, Kao J-H, Chao Y-C (2013). Interferon-based therapy reduces risk of stroke in chronic hepatitis C patients: a population-based cohort study in Taiwan. Aliment Pharmacol Ther.

[5] Hsu Y-C, Lin J-T, Ho HJ (2014). Antiviral treatment for hepatitis C virus infection is associated with improved renal and cardiovascular outcomes in diabetic patients. Hepatology.

[6] Dandel M, Lehmkuhl H, Knosalla C (2009). Strain and strain rate imaging by echocardiography – basic concepts and clinical applicability. Curr Cardiol Rev.

[7] Lang R, Badano L, Mor-Avi V (2015). Recommendations for cardiac chamber quantification by echocardiography in adults: an update from the American society of echocardiography and the European association of cardiovascular imaging. J Am Soc Echocardiogr.

[8] Schiller NB, Shah PM, Crawford M (2012). Recommendations for quantitation of the left ventricle by two-dimensional echocardiography. American society of echocardiography committee on standards, Subcommittee on quantitation of two-dimensional echocardiograms. J Am Soc Echocardiogr.

[9] Butz T, Van Buuren F, Mellwig KP (2011). Two-dimensional strain analysis of the global and regional myocardial function for the differentiation of pathologic and physiologic left ventricular hypertrophy: a study in athletes and in patients with hypertrophic cardiomyopathy. Int J Cardiovasc Imaging.

[10] Das D, Pandya M (2018). Recent advancement of direct-acting antiviral agents (DAAs) in hepatitis C therapy. Mini-Reviews Med Chem.

[11] Gomaa A, Allam N, Elsharkawy A (2017). Hepatitis C infection in Egypt: prevalence, impact and management strategies. Hepat Med.

[12] Shah A, Solomon S (2012). Myocardial deformation imaging: current status and future directions. Circulation.

[13] Kalam K, Otahal P, Marwick TH (2014). Prognostic implications of global LV dysfunction: a systematic review and meta-analysis of global longitudinal strain and ejection fraction. Heart.

[14] Motoki H, Borowski AG, Shrestha K (2012). Incremental prognostic value of assessing left ventricular myocardial mechanics in patients with chronic systolic heart failure. J Am Coll Cardiol.

[15] Zhang KW, French B, May Khan A (2014). Strain improves risk prediction beyond ejection fraction in chronic systolic heart failure. J Am Heart Assoc.

[16] Sengeløv M, Jørgensen PG, Jensen JS (2015). Global longitudinal strain is a superior predictor of all-cause mortality in heart failure with reduced ejection fraction. JACC Cardiovasc Imaging.

[17] Biomy R, Abdelshafy M, Abdelmonem A (2017). Effect of chronic hepatitis C virus treatment by combination therapy on cardiovascular system. Clin Med Insights Cardiol.

[18] Mazzitelli M, Torti C, Sabatino J (2018). Evaluation of cardiac function by global longitudinal strain before and after treatment with sofosbuvir-based regimens in HCV infected patients. BMC Infect Dis.

[19] Ahmad T, Yin P, Saffitz J (2015). Cardiac dysfunction associated with a nucleotide polymerase inhibitor for treatment of hepatitis C. Hepatology.

[20] El-Adawy A, Altonbary A, Hakim H (2018). Influence of different regimens of direct acting antiviral agents (DAAS) with or without ribavirin used for chronic hepatitis C treatment on the cardiac muscles in Egypt. J Med Res.

[21] Luo S, Rush R, Standring D (2016). Single- and repeat-dose toxicity of IDX14184, a nucleotide prodrug with antiviral activity for hepatitis C viral infection, in mice, rats, and monkeys. Hum Exp Toxicol.

[22] Hasenfuss G and Mann D (2018) Pathophysiology of heart failure. In D. Zipes, P. Lippy, R. Bonow (Eds.), Braunwald’s heart disease: a textbook of cardiovascular medicine (11th edition), Elsevir, Philadelphia 442-461.

[23] Ibrahim M, Sharafeldin A, Mousa N (2020). Effect of direct-acting antivirals on corrected QT interval and cardiac functions in patients with chronic hepatitis C virus infection. Egypt Hear J.

[24] Dawood A, Nooh M, Elgamal A (2017). Factors associated with improved glycemic control by direct-acting antiviral agent treatment in Egyptian type 2 diabetes mellitus patients with chronic hepatitis C genotype 4. Diabetes Metab J.

[25] Mada P, Malus M, Parvathaneni A et al (2020) Impact of treatment with direct acting antiviral drugs on glycemic control in patients with hepatitis C and diabetes mellitus. Int J Hepatol 2020:6438753. 10.1155/2020/6438753. 10.1155/2020/6438753 PMC7201615 32395351

[26] Zuo H, Zhang Y, Ma F (2020). Myocardial deformation pattern differs between ischemic and non-ischemic dilated cardiomyopathy: the diagnostic value of longitudinal strains. Ultrasound Med Biol.

